# The influence of chlorothalonil on the activity of soil microorganisms and enzymes

**DOI:** 10.1007/s10646-018-1968-7

**Published:** 2018-09-01

**Authors:** Małgorzata Baćmaga, Jadwiga Wyszkowska, Jan Kucharski

**Affiliations:** Department of Microbiology, University of Warmia and Mazury in Olsztyn, Plac Łódzki 3, Olsztyn, 10-727 Poland

**Keywords:** Chlorothalonil, Soil, Contamination, Fertilizing substances, Microbial and biochemical activity, Degradation

## Abstract

As one of the most widely used pesticides in agriculture, chlorothalonil can pose threat to soil ecosystems. Therefore, the impact of this substance on the development of microbiological and biochemical properties of the soil as well as on the growth of spring wheat was evaluated. The study was conducted with two soils (loamy sand with pH_KCl_ 5.6 and sandy loam with pH_KCl_ 7.00), to which fungicide was used in the following doses: 0.00, 0.166 (recommended dose), 1.660, and 16.60 mg kg^−1^ dry matter of soil (DM of soil). In addition, we determined the effectiveness of fertilizing substances (Lignohumat Super and Bioilsa N 12.5) in the restoration of soil homeostasis and chlorothalonil degradation in the soil. Chlorothalonil caused modifications in the count and biological diversity of soil microorganisms. It stimulated the growth of heterotrophic bacteria and actinobacteria, and inhibited the growth of fungi. This pesticide was a potent inhibitor of dehydrogenase, catalase and acid phosphatase activities. It showed variable effects on urease and alkaline phosphatase. The fungicide also a reduction the yield of dry matter of the aboveground parts of spring wheat. It should, however, be noted that these changes in the soil environment occurred after the introduction of higher doses of chlorothalonil. The fertilizing substances used contributed to enhanced microbial and biochemical activities of soils, while they did not significantly affect plant yields. The Bioilsa N 12.5 preparation was effective in chlorothalonil degradation, while Lignohumat Super reduced the degradation rate of the tested fungicide. Based on the conducted experiment, an ecological risk assessment of chlorothalonil was made by estimating the changes occurring in the soil environment evaluated through the microbiological and biochemical analyses of the soil.

## Introduction

The intensive development of agriculture and the increased food demand have led to massive applications of plant protection products. Fungicides are among the most important and most commonly used groups of pesticides, which are applied to protect plants against fungal diseases. They contain one or more active substances, the action of which in fungal cells includes: arrest of mitosis and cell division; inhibition of respiration, synthesis of nucleic acids, amino acids and proteins, lipids and cell membranes, as well as cell wall melanin, but also inhibition of sterol and cell wall biosynthesis, and signal transduction (Petit et al. 2012; Ijaz et al. 2015). Fungicides primarily allow obtaining high plant yields, but they can also exert negative effects on the environment, including the soil, by disrupting its homeostasis. Multiple applications of these preparations during the growing season make them not only effective in eliminating target organisms but also in destroying other non-targeted organisms. The accumulation of fungicides in the soil increases the risk of negative effects on organisms present in this ecosystem as well as on biological processes (Devashree et al. 2014). Microorganisms involved in soil-forming processes, organic matter transformation, stabilization of soil aggregates and the circulation of elements play an important role in maintaining adequate soil fertility. Interaction of soil microorganisms with other organisms (e.g. earthworms, nematodes, plants) present in the soil can significantly affect the quality and health of plants, and thus indirectly influence the level of agricultural production and sustainability of soil ecosystems (Shahgholi 2014).

Chlorothalonil is one of the most commonly used fungicides in the world. This is a synthetic compound from the group of chlorinated benzonitriles. It was first registered in the United States in 1966. In fungus cells, it leads to the inhibition of enzymes responsible for cellular respiration (Shi et al. 2011). Chlorothalonil is very effective in protecting plants against fungal diseases caused mainly by *Phytophthora infestans* and *Alternaria solani*, and prevents germination of fungal spores (Leitao et al. 2014). It is toxic to aquatic organisms (fish, invertebrates), birds, and also to humans, as it may cause inflammation of the skin, eyes and gastrointestinal disorders (Wang et al. 2011). Chlorothalonil is also considered a carcinogen (Wang et al. 2011). Leitao et al. (2014) reported its half-life to range from 0.3 to 87 days in the soil under laboratory conditions, while from 18 to 70 days in field conditions. According to Wang et al. (2011), the half-life decomposition of chlorothalonil in the soil ranged from 19 to 30 days. However, this substance can be detected in the soil for 100 days or even 1 year because it is often repeatedly applied during the growing season in some regions (Chaves et al. 2008). Due to its widespread use in crop plant protection, its residues are often detected in agricultural crops, fruits, vegetables, in the soil and water environment and in the air (Chaudhuri et al. 2013). Chlorothalonil can undergo chemical and microbiological degradation in the soil environment. Its degradation results in the formation of many metabolites : 4-hydroxy-2,5,6-trichloroisophthalonitrile, 1,3-dicarbamoyl-2,4,5,6-tetra chlorobenzene, 2,5,6-trichloro-4-methoxy isophthalonitrile, 1-carbamoyl-3-cyano-4-hydroxy2,5,6-trichlorobenzene, 2,4,5-trichloroisophthalonitrile, 2,5,6-trichloro-4 (methylthio)isophthalonitrile, isophthalonitrile (Chaves et al. 2008).

**Fig. 1:**
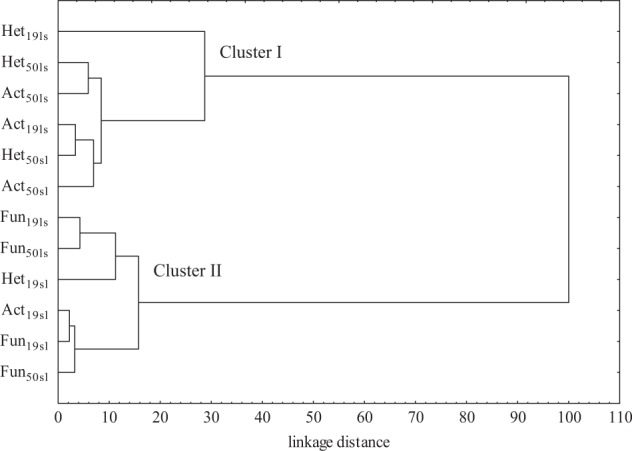
Similarity of microorganisms response to chlorothalonil soil contamination. Microorganisms: Het heterotrophic bacteria, Act actinobacteria, Fun fungi; type of soil: ls loamy sand, sl sandy loam; date of analysis: 19–19 days, 50–50 days

The negative effect of fungicides on the soil environment can be minimized by the use of suitable fertilizing substances such as compost, manure, straw or sewage sludge (Kadian et al. 2008; Pimmata et al. 2013). Soil supplementation with such substances often leads to the stimulation of soil microorganisms that may exhibit the ability to degrade fungicides present in this environment (Adams et al. 2015).

The use of fungicides may pose serious risk to the natural environment surrounding us. It may be manifested by the disruption of the correct structure and functions of soil ecosystems. According to Saha et al. (2016) and Sułowicz and Piotrowska-Seget 2016, literature still lacks detailed information on the effects of active substances of fungicides on the biological properties of soil. Due to the risk of soil ecosystems contamination with fungicides, this study was conducted to determine the impact of chlorothalonil on the biological properties of the soil. In addition, the efficacy of Lignohumat Super and Bioilsa N 12.5 fertilizing substances was evaluated in homeostasis restoration of soil treated with chlorothalonil.

## Materials and methods

### Soil material

Soil material was derived from the Didactic and Experimental Center in Tomaszków near Olsztyn, Poland. The characteristics of the area have been given in the work of Borowik et al. (2017). Soils used in the experiment belonged to Eutric Cambisols, and in terms of texture were classified as loamy sand and sandy loam. The physicochemical properties of the soils given in Table 1 were determined according to methods described by Carter (1993).

**Table 1:** General characteristics of experimental soil

Type of soil	Sand (50–2000 µm)	Silt (2−50 µm)	Clay < 2 µm	C_org_	N_total_	pH_KCl_	HAC	EBC	CEC	BS%
	%			g kg^−1^ DM of soil				mMol	(+ ) kg^−1^DM of soil	
Loamy sand	80.50	18.00	1.50	10.00	0.58	5.60	18.66	40.00	58.66	68.19
Sandy loam	72.00	21.00	7.00	7.05	0.86	7.00	8.000	111.0	119.00	93.28

*C*_*org*_ organic carbon content, *N*_*total*_ total nitrogen content, *HAC* hydrolytic acidity, *EBC* exchangeable base cations, *CEC* cation exchange capacity, *BS* base saturation

### Fungicide

Gwarant 500 SC is a contact fungicide designed to protect wheat, potato, tomato and cucumber against fungal diseases. It is a concentrate in a suspension for dilution with water, produced by Arysta LifeScience, which was authorized for marketing in Poland in 2011. In wheat protection, it is applied at a dose of 1.0 dm^3^ ha^−1^, 1 dm^3^ of this preparation contains 500 g chlorothalonil, which is one of the compounds from the group of chloronitriles. Considering that, 0.166 mg of chlorothalonil was applied per 1 kg of soil.

### Soil fertilizer amendments

Lignohumat Super and Bioilsa N 12.5 fertilizing substances were introduced at the manufacturer’s recommended doses due to possible negative effects of the chlorothalonil on the biological properties of the soil. Lignohumat Super is a substance produced by Agrarius. It contains 90% of humic substances (including 80–85% salts of humic acids and 15–20% of fulvic acids) and 10% of macronutrients and micronutrients (9% potassium, 3% sulfur, 0.02% iron, 0.12% copper, 0.012% manganese, 0.12% zinc, 0.15% boron, 0.15% molybdenum, 0.12% cobalt, trace amounts of calcium and silicon). The dose recommended by the manufacturer of this preparation is between 0.5 and 1.0 kg ha^−1^. In the experiment, Lignohumat Super was used at a dose of 0.333 mg kg^−1^.

In turn, Bioilsa N 12.5 is a controlled-release nitrate fertilizer for the fertilization of orchard, vegetable, and agricultural plants. It is produced by Natural Crop and contains: organic nitrogen—12.5% (including dissolved organic nitrogen—5%), organic carbon of biological origin—40% (including extractable organic carbon—95%), and organic matter—70%. The dose recommended by the manufacturer for cereals ranges between 400 and 700 kg ha^−1^. In the experiment, this preparation was applied at a dose of 233 mg kg^−1^.

### Procedure and conditions of the experiment

The pot experiment was performed in five replicates in polyethylene pots of 3.5 dm^3^ volume containing 2.7 kg of loamy sand with pH_KCl_ 5.6 and sandy loam with pH_KCl_ 7.0. On the day of starting the experiment, CaCO_3_ was added to loamy sand (pH_KCl_ 5.6) in the amount equilibrating the hydrolytic acidity at the level of 1.5 HAC (hydrolytic acidity) to neutralize the soil. The Gwarant 500 SC fungicide was used in the experiment, which, was used at the following doses (based on the active substance): 0 (soil without fungicide addition), 0.166 (manufacturer’s recommended dose), 1.660 (10-fold the recommended dose), and 16.60 mg kg^−1^ (100-fold the recommended dose). The aqueous solution of the fungicide was applied; 1 cm^3^ of the solution per 1 kg dry matter of soil. Three water solution were prepared on the day when the experiment had been established and applied once in the tested doses to the soil. 1 cm^3^ of particular solution contained: 0.333 mm^3^ (optimal dose), 3.33 mm^3^ (10-fold higher dose), and 33.33 mm^3^ (100-fold higher dose) of the fungicide. Mineral fertilizer doses were applied to the soil in the following quantities (calculated per pure component in mg kg^−1^): N–100 (urea), P–44 (potassium dihydrogen phosphate), K–100 (potassium dihydrogen phosphate_ + _potassium chloride), Mg–25 (magnesium sulfate heptahydrate). Lignohumat Super and Bioilsa N 12.5 fertilizing substances were introduced at the manufacturer’s recommended doses, due to possible negative influences of the fungicide on the biological activity of the soil. The fertilizing substances were introduced once to the soil material on the day of experiment establishment. Lignohumat Super was used in the form of a water suspension prepared from a granulate. 1 cm^3^ of the water solution contained 0.333 mg of the tested fertilizing substance. In turn, Bioilsa N 12.5 was added to soil in the form of a granulate in weighted portions of 233 mg kg^−1^. The tested crop was spring wheat of cultivar *Arabella*. The soil moisture was maintained at 50%. Soil humidity was maintained at a stable level by its continuous monitoring. Its level was checked every day and adjusted with distilled water. The vegetation pot experiment was conducted in polyethylene pots in five replications for each combination (giving a total of 120 pots): four doses of chlorothalonil, two types of soil, three types of fertilizers, five replications. Before analyses made on two sampling moments (day 19 and 50), soil samples were collected from each combination (from five polyethylene pots for each combination) and mixed into a collective sample of 500 g that was thoroughly homogenized. To obtain samples, the soil material was sampled from each pot across its whole depth. Thus, prepared collective samples were used for determinations of the microbiological and biochemical properties of soil, that were made in three replications for each sample. Residues of chlorothalonil were determined on day 19 in soil specimens with the highest fungicide dose (16.60 mg kg^−1^), while the yield of spring wheat on day 50.

### Microbiological analyses

Microbiological analyses were performed in three replicates at two experimental time points (day 19 and 50) using the plate method. The count of actinobacteria, fungi, and heterotrophic bacteria was determined according to the procedure described in the work of Kucharski et al. (2016). Observations of microbial colony growth were carried out for 9 days at 3-day intervals, when the count of microorganisms was determined. Based on the count of microorganisms, the count of microbial colonies growing at certain time intervals (K_s_) was calculated, according to the formula given in the work by Tomkiel et al. (2015).

### Enzymatic analyses

The enzymatic activity of soil was determined on 19 day and 50 day after the start of the experiment. The biochemical analyses included determination of the activities of: acid phosphatase, alkaline phosphatase, catalase, dehydrogenases, and urease. To determine the activity of soil enzymes, the samples were incubated in a thermostat (with no access to light) at a temperature of 37 ^o^C. The activity of soil enzymes, with the exception of catalase, was determined using a Perkin-Elmer Lambda 25 (Massachusets, USA) spectrophotometer. The exact methodology for determining the activity of soil enzymes is provided in the paper of Borowik et al. (2017).

### Determination of chlorothalonil residues in the soil

Residues of chlorothalonil in soil samples were determined with the GC-EC method (gas chromatography with an electron capture detector). Chlorothalonil content in soil samples was determined using an Agilent 7890 A (Santa Clara, Kafifornia, USA). Data were received and processed by the Chemstation (Hewlett-Packard, version A.10.2) software. Quantitative determination was carried out by comparing peak heights obtained in soil samples against the standards used (Łozowicka et al. 2016). Chlorothalonil detection limit reached 0.005 mg kg^−1^, whereas analytical recovery value ranged from 70 to 75%.

### Spring wheat development

Spring wheat, cultivar *Arabella*, was used as a test plant to evaluate chlorothalonil phytotoxicity. After 7 days from the start of the experiment, spring wheat was sown in the pots in the quantity of 25 seeds per pot. When the spring wheat was sprouting (BBCH 10—the first leaf emerges from the leaf sheath), a thinning was performed, leaving 12 plants in the pot. Soil moisture was maintained at 50% throughout the vegetation period. The plants according to the BBCH scale (scale of plant growth stage) were harvested at the end of the heading stage of spring wheat (BBCH 59—all spikelets emerge from the sheath, the head is completely visible). For this purpose, the above-ground parts were cut and then dried at a temperature of 65^ o^C for 5 days. After thorough drying of the plant material, the dry plant biomass was determined on the basis of which the resistance (RS) of spring wheat in the soil treated with chlorothalonil was calculated according to the formula described by Orwin and Wardle (2004).

### Statistics

The statistical analyzes were performed using Statistica 12.5 software (Statsoft, Inc, Statistica 2017). The *η*^2^ coefficient was used to calculate the percentage of observed variability of the analyzed factors (ANOVA at a significance level of *P* < 0.05). Pearson’s linear correlation coefficients were calculated between the variables studied. Homogeneous groups were determined (*P* < 0.05) by the Tukey’s test using ANOVA variance analysis. The responses of microorganisms to chlorothalonil action and soil fertilizing substances were presented as a Ward dendrogram using cluster analysis. In turn, the activity of soil enzymes was demonstrated by principal component analysis.

## Results and discussion

### Soil microorganisms

**Fig. 2:**
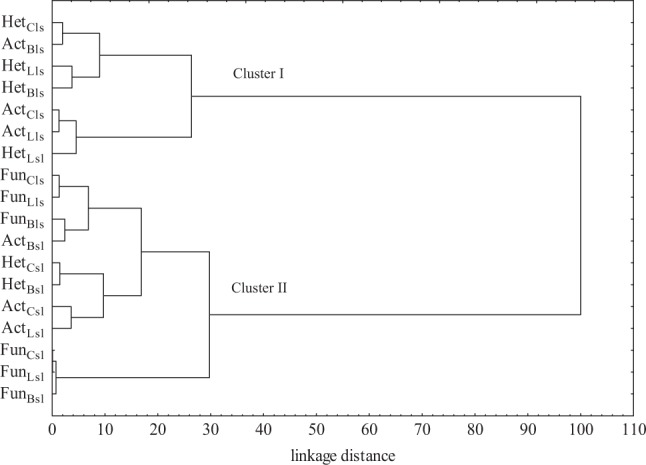
Similarity of microorganisms response to fertilizing substance. Microorganisms: Het heterotrophic bacteria, Act actinobacteria, Fun fungi; type of soil: ls loamy sand, sl sandy loam; fertilizing substance: C soil without fertilizing substance addition, L soil with the addition of Lignohumat Super, B soil with the addition of Bioilsa N 12.5

In the current study, the analysis of variance (Table 2) showed that the count of microorganisms was most influenced by the soil type (heterotrophic bacteria in 39%, actinobacteria in 49%, and fungi in 54%). Chlorothalonil applied to sandy loam and loamy sand caused changes in the count of soil microorganisms (Table 3). The fungicide introduction at doses from 0.166 to 16.60 mg kg^−1^ in loamy sand stimulated the growth of heterotrophic bacteria and actinobacteria both on day 19 and 50 of the experiment. On day 19 of the experiment, chlorothalonil caused an increase in the count of heterotrophic bacteria, which ranged from 27% (16.60 mg kg^−1^) to 98% (0.166 mg kg^−1^), whereas on day 50 of the experiment—an increase in heterotrophs count from 22% (0.166 mg kg^−1^) to 47% (16.60 mg kg^−1^). The count of actinobacteria in the pots exposed to the tested fungicide increased from 27% (0.166 mg kg^−1^) to 43% (16.60 mg kg^−1^) on day 19 as well as from 10% (0.166 mg kg^−1^) to 64% (16.60 mg kg^−1^) on day 50 of the experiment, compared to the control. In the case of fungi, a significant decrease in their count was observed with respect to the control sample. However, the greatest changes were caused by fungicide dose of 16.60 mg kg^−1^ (fungi count decreased by 76% on day 19 and by 35% on day 50). In sandy loam, the count of heterotrophic bacteria increased at both experimental time points of measurements. A slight differences was observed in actinobacteria and fungi response to the test preparation. It was recorded that on day 19, the count of actinobacteria and fungi decreased compared to the control soil (Table 3). After 50 days since chlorothalonil application in doses ranging from 0.166 to 16.60 mg kg^−1^, its stimulating effect was observed on counts of actinobacteria and fungi. Considering the mean values, the abundance of heterotrophic bacteria at the highest fungicide dose (16.60 mg kg^−1^) increased by 36% in loamy sand and by 53% in the sandy loam, compared to the control sample. Regarding actinobacteria, the same dose increased the abundance of these microorganisms by 54% and 79%, respectively. In loamy sand, a reduction in the count of fungi was noted with an increase in the fungicide dose. In sandy loam, a fungicide dose of 1.660 mg kg^−1^ increased the count of fungi by 30.7% compared to the soil without fungicide addition. Other doses did not significantly affect fungal growth. Considering that the soil environment is characterized by a vast species diversity of microorganisms (including fungi), it is likely that the soil could be colonized by fungi resistant to chlorothalonil. Intensive farming methods, based on chemical plant protection, can contribute to fungicide penetration to the soil. These substances present in the soil environment pose a potential threat to non-target organisms, including microorganisms. Soil-dwelling microorganisms play significant functions in the nutrient cycling as well as in soil-forming processes. They also enhance plants growth by secreting, among others, enzymes, hormones, and siderophores facilitating iron binding. They can also participate in biological protection of plants against pathogens. Changes in the soil environment caused by the presence of fungicides usually lead to a reduction in the abundance and diversity of microorganisms. The impact of these preparations on soil-dwelling microorganisms depends primarily on the chemical structure of the fungicide itself and its dose, as well as the soil type (Handsa et al. 2014). Hence, organic matter plays a significant functions in protecting the soil environment against chemical contamination. Its high content in the soil affects the sorption properties. In addition, organic matter is capable to adsorb contaminants, including pesticides, which accelerates their degradation (Banach-Szott et al. 2014). Organic compounds are sources of C, N, and P. They are used by microorganisms as nutrients and energy sources. Soils rich in organic matter also have a higher biological activity (Wang et al. 2009). The cluster analysis (CA) shown in Fig. 1 also indicated the various response of soil microorganisms to chlorothalonil. The first cluster was formed by heterotrophic bacteria and actinobacteria isolated from loamy sand and sandy loam on day 50, and heterotrophic bacteria and actinobacteria from loamy sand on day 19. Counts of these groups of microorganisms were observed to increase along with an increasing dose of chlorothalonil added to the soil. Of all the microorganisms present within this cluster, heterotrophic bacteria isolated from sandy loam on day 50 and actinobacteria isolated from loamy sand on day 19 showed the most similar response to chlorothalonil. The second cluster consisted of heterotrophic bacteria and actinobacteria isolated from sandy loam on day 19 and fungi isolated from loamy sand and sandy loam on days 19 and 50 of the experiment. Based on the clusters formed, it can be stated that actinobacteria and heterotrophic bacteria reacted similarly to the fungicide introduced to the soil. These microorganisms responded to fungicide presence in the soil with their populations increase. It should be noted that the greatest similarity of microorganism response to chlorothalonil action was found between fungi and actinobacteria in sandy loam on day 19. Many studies (Garcίa-Gil et al. 2013; Mohiuddin and Mohammed 2013) indicated that fungicides may lead to the destabilization of soil ecosystems by decreasing the abundance of microorganisms. Studies by Yu et al. (2006) on chlorothalonil have shown that, when introduced into the soil, this substance can have a negative effect on the population of bacteria, fungi, and actinobacteria. The response of soil microorganisms to the applied fungicide Amistar 250 SC (a.s. azoxystrobin) was also negative in the study conducted by Baćmaga et al. (2015). The toxic effects of benomyl, carbendazim, carboxin, captan, cyclohexamide, fenpropimorph, propiconazole, and triam fungicides on the bacterial population were observed by Milenkovski et al. (2010). In turn, Guo et al. (2015) demonstrated an inhibiting effect of azoxystrobin on the proliferation of bacteria, actinobacteria and fungi, and these changes were dependent on the dose and the retention of the substance in the soil. These authors noted that azoxystrobin applied at doses from 0.1 to 10 mg kg^−1^ caused the greatest changes in the microbial population after 7 days of soil incubation, i.e., on days 14, 21, and 28. In our study, the introduction of soil fertilizing substances also affected microbiological properties of the soil, thereby contributing to changes in the counts of the analyzed soil microorganisms (Table 3). The average results indicated that the application of Lignohumat Super and Bioilsa N 12.5 to the soil caused an increase in the count of heterotrophic bacteria in both loamy sand and sandy loam, however, a larger impact was observed after the addition of Lignohumat Super. This substance contains as much as 90% humic acids, which are the source of nutrients, vitamins, and micronutrients. In addition, humic acids also improve soil structure and prevent loss of water and nutrients (Arjumend et al. 2015). Therefore, an increase in the count of microorganisms in the soil could be due to humic acids present in the preparation, which ensured favorable conditions for their development (Hale and Fawy 2011). The application of Bioilsa N 12.5 stimulated the growth of actinobacteria in both loamy sand and sandy loam. The tested fertilizing substances contributed also to an increased count of fungi. Taking into account the term of analysis, it was observed that after Lignohumat Super had been introduced to both loamy sand and sandy loam, the average count of heterotrophic bacteria was the highest on day 19, whereas that of actinobacteria and fungi on day 50. The effect of Bioilsa N 12.5 on microorganisms counts was slightly different over time, i.e. in loamy sand the microorganisms were more abundant on day 19, while in sandy loam on day 50. The similarity of microorganisms responses to fertilizing substance addition to the soil is shown in Fig. 2. Two groups of microbial responses to the fertilizing substances were identified based on the created dendrogram. Two subgroups were distinguished in the first group, while three subgroups were identified in the second group. The microbial response was determined not only by the type of fertilizing substance, but also by the soil type. However, the highest similarity in response to the fertilizing substances was found in sandy loam.

**Table 2:** The percentage of the observed *η*^2^ variability of the effect of the analyzed factors on soil microorganisms (four-way analysis of variance, ANOVA, at *P* < 0.05)

Variables	Het	Act	Fun
D	7.494	1.456	11.013
S	39.110	48.934	53.713
Cs	3.886	1.357	5.608
T	3.934	6.508	2.636
D × S	0.951	0.406	5.349
D × Cs	4.787	2.925	1.797
S × Cs	0.166	1.888	6.468
D × T	0.350	1.106	2.918
S × T	17.108	9.328	1.439
Cs × T	3.664	6.162	0.374
D × S × Cs	1.707	4.454	2.388
D × S × T	2.235	4.378	0.837
D × Cs × T	2.982	3.758	1.318
S × Cs × T	1.182	1.121	0.492
D × S × Cs × T	4.705	2.802	0.657
Error	5.738	3.416	2.994

*Het* heterotrophic bacteria, *Act* actinobacteria, *Fun* fungi, *D* dose of fungicide, *S* type of soil, *Cs* fertilizing substance, *T* date of analysis

**Table 3:** The count of soil microorganisms, 10^n^ cfu kg^−1^ DM of soil

Dose of fungicide (mg kg^−1^)	Loamy sand						Sandy loam					
	Het × 10^9^		Act × 10^9^		Fun × 10^7^		Het × 10^9^		Act × 10^9^		Fun × 10^7^	
	Date of analysis (days)											
	19	50	19	50	19	50	19	50	19	50	19	50
Soil without fertilizing substance addition												
0.000	20.064^b^	16.121b	14.638^d^	16.247^cd^	8.991^ab^	6.972^bc^	5.951^d^	14.892^bc^	2.393^c^	10.800^b^	2.172^c^	2.141c
0.166	39.747a	19.638b	18.664^bc^	17.815^bcd^	5.027^cd^	9.395^a^	10.011^cd^	17.962^ab^	2.376^c^	14.066^b^	2.059^c^	2.281c
1.660	28.086ab	22.782b	20.241^b^	19.017^bc^	4.958^cd^	5.962^cd^	13.687^bc^	18.291^ab^	2.819^c^	15.504^b^	0.940d	4.700a
16.60	25.439b	23.658b	21.001^b^	26.720^a^	2.125^e^	4.500^d^	11.771^c^	20.170^a^	1.654^c^	21.919^a^	0.509d	3.563b
Average	28.334	20.550	18.636	19.950	5.275	6.707	10.355	17.829	2.310	15.572	1.420	3.171
*r*	−0.233	0.669	0.613	0.985	−0.778	−0.755	0.365	0.753	−0.863	0.934	−0.797	0.307
Lignohumat Super												
0.000	34.453a	26.779^ab^	12.979^c^	22.295^ab^	8.147^ab^	5.968^bc^	25.322^a^	14.339^b^	11.332^bc^	9.306^cd^	2.184bcd	3.165b
0.166	34.476a	30.068^ab^	14.767^bc^	21.693^ab^	6.801^b^	9.886^a^	19.124^ab^	17.438^b^	5.537de	12.700^bc^	1.591cd	5.314a
1.660	34.794a	19.613^b^	26.465^a^	20.556^abc^	6.286^bc^	5.783^bc^	16.765^b^	16.415^b^	5.493de	13.907^b^	1.588cd	2.604bc
16.60	40.890^a^	15.681^b^	14.230^bc^	16.911^bc^	6.216^bc^	3.660^c^	15.350^b^	15.063^b^	4.108e	22.515^a^	0.732d	1.656^bcd^
Average	36.153	23.035	17.110	20.364	6.862	6.324	19.140	15.814	6.617	14.606	1.524	3.185
*r*	0.999	−0.800	−0.216	−0.977	−0.543	−0.712	−0.630	−0.336	−0.565	0.959	−0.905	−0.696
Bioilsa N 12.5												
0.000	23.680^cd^	18.034^cd^	23.272^b^	22.519^bc^	14.929^a^	13.424a	10.225^de^	18.029^ab^	6.180c	14.270^a^	0.796d	3.026b
0.166	28.575^bc^	24.138^cd^	23.414^b^	18.945^cd^	10.480^bc^	12.746ab	15.686^bc^	18.518^ab^	11.518b	11.518^b^	1.082d	5.441a
1.660	41.665^a^	24.540^bcd^	23.472^b^	18.664^d^	7.981^cd^	8.044cd	12.726^cd^	20.184^a^	9.616b	10.822b	1.269cd	3.047b
16.60	35.428^ab^	15.107^d^	34.701^a^	17.350^d^	6.510^d^	5.752d	8.034^e^	19.480^ab^	2.837d	10.043b	0.287d	2.518^bc^
Average	32.337	20.455	26.215	19.370	9.975	9.991	11.668	19.053	7.538	11.663	0.859	3.508
*r*	0.349	−0.727	0.997	−0.652	−0.684	−0.820	−0.730	0.382	−0.800	−0.639	−0.851	−0.534

*Het* heterotrophic bacteria, *Act* actinobacteria, *Fun* fungi, *r* Pearson’s linear correlation coefficient Homogeneous groups were marked with the same letters separately for each group of microorganisms, the type of the soil and the type of fertilizing substance

Fungicide penetration to the soil can also lead to changes in the structure and diversity of soil microbial communities (Imfeld and Vuilleumier 2012). Biodiversity of soil microorganisms is very important, in terms of sustaining the proper soil health, and therefore, improved crop yields. Disturbance of the activity of soil microorganisms may affect the nutritive soil properties (Janvier et al. 2007). In the current study, the emergence rate of microorganism colonies was largely dependent on soil contamination with chlorothalonil (Tables 4, 5). The average results from the two experimental time points indicated that the highest count of fungi and heterotrophic bacteria was found in the first three days of microbial culture in both loamy sand and sandy loam, which were not exposed to chlorothalonil. This indicated the presence of rapidly growing microorganisms that exhibited adaptive capacity under unfavorable environmental conditions and developed fungicide resistance mechanisms. As a result, microorganisms were able to degrade fungicides by using them as a source of nutrients (Shahgholi 2014). The colonies count of actinobacteria was recorded after 6 days (loamy sand—42% and sandy loam—41%). The count of colonies of heterotrophic bacteria in loamy sand contaminated with chlorothalonil varied from 8 to 68%, that of actinobacteria from 27 to 40%, and that of fungi from 4 to 85%. In sandy loam, heterotrophic bacteria developed in the range from 5 to 76%, actinobacteria from 23 to 40% and fungi from 10 to 83%. Changes in the structure of microorganisms under the influence of triadimefon and propiconazole introduced into the soil at doses of 10 and 100 mg kg^−1^ were recorded by Yen et al. (2009). In turn, Wang et al. (2009) demonstrated that the repeated application of carbendazim (at doses from 0.94 to 4.70 kg a.i ha^−1^) could reduce the diversity of the bacterial population and alter the structure of their community. Most colonies of heterotrophic bacteria and fungi in the soil supplemented with Lignohumat Super grew after 3 days of the culture. In this period, on average 65% of colonies of heterotrophic bacteria and 68% of fungi grew in loamy sand as well as 73% of colonies of heterotrophic bacteria and 89% of fungi in sandy loam. In the objects with Biolilsa N 12.5 addition, colonies of all analyzed microorganisms groups grew fastest on day 3 of the culture (heterotrophic bacteria amounted on average to 66%, actinobacteria—39% and fungi—79%). In sandy loam, the highest count of grown colonies of heterotrophic bacteria and fungi was recorded on day 3 of the culture (57% and 66%, respectively).

**Table 4:** The average count of microorganism colonies (%) growing at particular time intervals (K_s_) in loamy sand at the two time points

Dose of fungicide (mg kg^−1^)	Het			Act			Fun		
	Days of culture								
	3 day	6 day	9 day	3 day	6 day	9 day	3 day	6 day	9 day
Soil without fertilizing substance addition									
0.000	54.592b	34.781c	10.627e	30.354cd	41.824a	27.822c	83.136ab	12.145e	4.718f
0.166	65.750a	25.505cd	8.745e	33.112bcd	40.239ab	26.649c	85.145a	10.399e	4.457f
1.660	68.117a	23.310d	8.573e	33.483c	39.836ab	26.681c	80.291b	11.722e	7.987ef
16.60	67.718a	24.692cd	7.590e	27.177	36.398abc	36.424abc	70.841c	17.030d	12.128e
Average	64.044	27.072	8.884	31.031	39.574	29.394	79.854	12.824	7.323
*r*	0.442	−0.366	−0.720	−0.846	−0.948	0.983	−0.971	0.967	0.931
Lignohumat Super									
0.000	64.921a	27.753b	7.326c	28.663fg	35.427cde	35.910bcde	71.748ab	17.853de	10.399ef
0.166	63.059a	27.018b	9.923c	23.649gh	35.574bcde	40.777ab	62.371bc	19.081de	18.549de
1.660	66.981a	23.182b	9.837c	20.840h	37.887abc	41.273a	60.888c	21.189d	17.923de
16.60	64.964a	23.940b	11.096c	32.515def	36.591abcd	30.895ef	78.778a	15.092def	6.129f
Average	64.981	25.473	9.545c	26.417	36.370	37.214	68.446	18.304	13.250
*r*	0.070	−0.535	0.687	0.731	0.221	−0.834	0.781	−0.789	−0.755
Bioilsa N 12.5									
0.000	66.036a	23.135b	10.829c	36.468bc	34.455cd	29.077de	80.494a	14.972b	4.534c
0.166	65.394a	23.754b	10.851c	41.345ab	36.543bc	22.112f	78.038a	17.564b	4.397c
1.660	64.069a	25.450b	10.482c	44.647a	33.946cde	21.407f	79.336a	17.005b	3.659c
16.60	69.764a	23.010b	7.226c	34.081cde	38.387abc	27.532ef	78.060a	18.792b	3.148c
Average	66.316	23.837	9.847	39.135	35.833	25.032	78.982	17.083	3.935
*r*	0.908	−0.409	−1.000	−0.649	0.801	0.378	−0.525	0.738	−0.858

*Het* heterotrophic bacteria, *Act* actinobacteria, *Fun* fungi, *r* Pearson’s linear correlation coefficient Homogeneous groups were marked with the same letters separately for each group of microorganisms and the type of fertilizing substance

**Table 5:** The average count of microorganism colonies (%) growing at particular time intervals (K_s_) in sandy loam at the two time points

Dose of fungicide (mg kg^−1^)	Het			Act			Fun		
	Days of culture								
	3 day	6 day	9 day	3 day	6 day	9 day	3 day	6 day	9 day
Soil without fertilizing substance addition									
0.000	72.330a	21.150b	6.520cd	30.655bcde	40.813a	28.532cde	81.271a	17.734cd	0.995e
0.166	63.875a	28.946b	7.179cd	26.412de	34.819abcd	38.769ab	74.044a	18.019cd	7.937d
1.660	65.620a	27.755b	6.621cd	26.497de	34.942abcd	38.561abc	60.885b	27.745c	11.370d
16.60	75.697a	19.471bc	4.832c	39.992ab	37.261abc	22.747e	82.702a	15.138d	2.160e
Average	69.381	24.331	6.288	30.889	36.959	32.152	74.725	19.659	5.616
*r*	0.728	−0.653	−0.962	0.930	0.019	−0.760	0.455	−0.463	−0.402
Lignohumat Super									
0.000	71.735ab	20.428cd	7.837fg	38.028ab	39.191ab	22.781cd	87.119a	11.086b	1.795c
0.166	73.427ab	18.075cde	8.498efg	40.254ab	39.518ab	20.228d	90.497a	5.075bc	4.428bc
1.660	77.637a	16.307def	6.056g	41.547a	37.469ab	20.984cd	89.057a	5.652bc	5.291bc
16.60	67.782b	27.050c	5.167g	35.247b	38.385ab	26.368c	88.549a	6.272bc	5.179bc
Average	72.645	20.465	6.890	38.769	38.641	22.590	88.806	7.021	4.173
*r*	−0.735	0.900	−0.799	−0.806	−0.274	0.905	−0.106	−0.227	0.473
Bioilsa N 12.5									
0.000	62.728a	30.244c	7.029d	36.801a	35.553a	27.646b	75.803a	16.689cd	7.509d
0.166	57.628ab	32.977c	9.395d	35.932a	29.277b	34.790a	54.171ab	35.818c	10.011d
1.660	55.121ab	34.603c	10.276d	35.154a	30.822b	34.024a	64.208ab	26.744c	9.049d
16.60	51.562b	34.669d	13.769d	28.356b	37.843a	33.801a	70.244a	17.162cd	12.594cd
Average	56.760	33.123	10.117	34.061	33.374	32.565	66.106	24.103	9.791
*r*	−0.786	0.563	0.903	−0.995	0.719	0.293	0.285	−0.498	0.882

*Het* heterotrophic bacteria, *Act* actinobacteria, *Fun* fungi, *r* Pearson’s linear correlation coefficient Homogeneous groups were marked with the same letters separately for each group of microorganisms soil and the type of fertilizing substance

### Soil enzymes

**Fig. 3:**
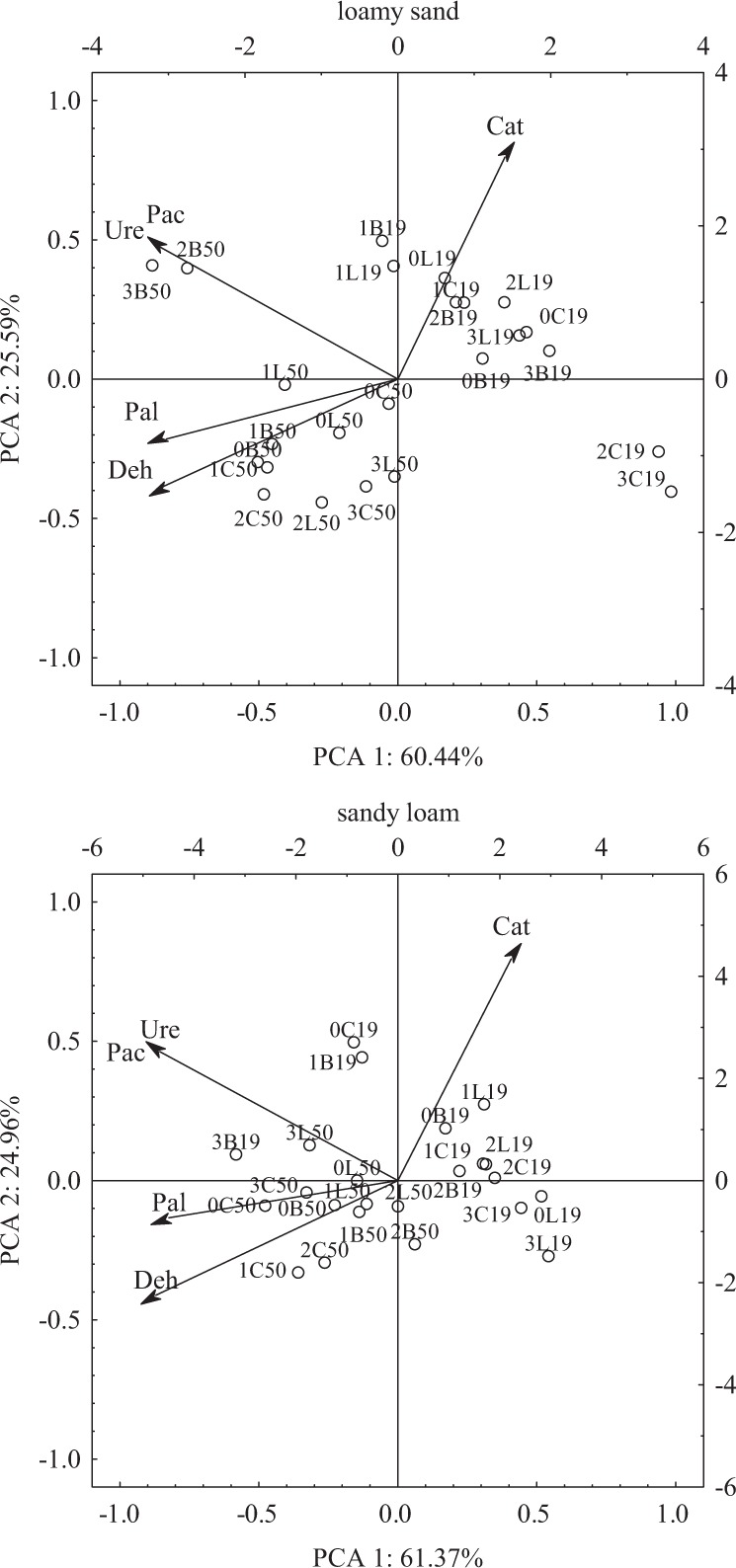
Enzyme activities in the soil treated with chlorothalonil with the addition of fertilizing substance. Enzymes: Deh dehydrogenases, Cat catalase, Ure urease, Pac acid phosphatase, Pal alkaline phosphatase; dose of fungicide (mg kg^−1^ DM of soil): 0 soil without the addition of fungicides, 1–0.166 mg, 2–1.660 mg, 3–16.60 mg; fertilizing substance: C soil without fertilizing substance addition, L soil with the addition of Lignohumat Super, B soil with the addition of Bioilsa N 12.5; date of analysis: 19–19 days, 50–50 days

Statistical analysis showed that the factors tested contributed to changes in the soil enzymes activity. Chlorothalonil dose affected mostly the activity of urease. Soil type had a stronger effect on the activities of acid phosphatase and alkaline phosphatase, whereas date of analysis—on the activities of dehydrogenase and catalase (Table 6). Any contaminant entering the soil can interfere with biogeochemical processes. Some fungicides can inhibit, but also stimulate the soil microbial activity. Disturbances in soil microbial activity indirectly affect the enzymatic activity of the soil ecosystem (Stefani et al. 2012). Suppression of the enzymatic activity of soil may be due to increased mortality of microorganisms triggered by toxic doses of pesticides. As a result of this, physiological processes of microorganisms are rejected. This in turn leads to modification of the course of enzymes activity, which are mainly secreted by microorganisms. By taking part in many processes proceeding in soil and in biogeochemical circulations, soil enzymes are reliable indicators of changes occurring in soil that may be potentially used to evaluate soil quality and fertility (Bielińska and Pranagal 2007; Kızılkaya et al. 2012; Ye et al. 2018). In line with this, results obtained in our study enable concluding that the analyzed factors exerted various effects on the biochemical properties of loamy sand (Table 7) and sandy loam (Table 8). Chlorothalonil addition to the soil in the highest dose (16.60 mg kg^−1^) had an inhibiting effect on activities of dehydrogenases, catalase, and acid phosphate in both soil types. In loamy sand, this dose of chlorothalonil suppressed the activity of dehydrogenases by 47%, that of catalase by 34%, and that of acid phosphatase by 17% on average, whereas in sandy loam—by 35%, 20%, and 34% in the case of dehydrogenases, catalase, and acid phosphatase, respectively. The response of the other enzymes to chlorothalonil was not that explicit and depended to a greater extent on soil type and date of analysis. The PCA analysis presented in Fig. 3 also confirmed the significant impact of the examined factors. The activity of soil enzymes in loamy sand was affected by the first principal component in 60% and by the second in 26%. Similar correlations were observed in sandy loam, where the first principal component explained 61.37% of the total variables variance, while the second 25%. The activity of soil enzymes was altered by the excessive amounts of chlorothalonil. Both in loamy sand and sandy loam (day 19 and 50), this fungicide inhibited the activity of acid phosphatase, catalase and dehydrogenases. The highest dose caused the greatest changes (16.60 mg kg^−1^). Chlorothalonil exerted variable effects on urease and alkaline phosphatase activities. This was primarily dependent on the type of the soil and the time point of analysis. Wu et al. (2012) observed that multiple applications of chlorothalonil at 10 mg and 25 mg kg^−1^ increased the stability of this substance, and thus led to changes in soil enzymatic activity. Sopeña and Bending (2013) tested three fungicides (azoxystrobin, chlorothalonil, tebuconazole) and reported lower dehydrogenase activity. The negative impact of Falcon 460 EC fungicide on soil enzymes (acid phosphatase, alkaline phosphatase, catalase, urease) was also reported in our previous study (Baćmaga et al. 2016), especially upon the use of the highest fungicide dose (27.6 mg kg^−1^). Tebuconazole used at 5, 50 and 500 mg kg^−1^ also reduced the activity of alkaline phosphatase, arylsulfatase, β-glucosidase and urease in the soil tested by Muñoz-Leoz et al. (2011). Saha et al. (2016) also investigated the effect of tebuconazole on soil enzymatic activity. They observed short-term and transient toxic effects of tebuconazole applied at three doses (dose recommended, twofold and tenfold higher doses than recommended) on the activity of arylsulfatase, fluorescein diacetate, phosphatase, and urease. However, the activity of dehydrogenases and nitrate reductase drastically decreased after application of this fungicide. A reduction in the activity of phosphatases, amylase, and invertase (a dose from 10 to 2000 mg kg^−1^) caused by mancozeb was reported by Walia et al. (2014). It should be emphasized that the negative impact of fungicides on the microbiological and biochemical properties of soil ecosystems is generally recorded in soils where these substances are applied at doses many times higher than those recommended by the manufacturer. Some preparations introduced into the soil in excess contribute to the stimulation of enzyme activity. Increases in dehydrogenase, arylamidase, and myrosinase activities were reported in response to the application of mancozeb and carbendazim (Srinivasulu and Rangaswamy 2013) fungicides to the soil. Prochlorazole also acted as an activating agent on dehydrogenase, urease, phosphatase, and β-glucosidase (Tejada et al. 2011).

**Table 6:** The percentage of the observed *η*^2^ variability of the effect of the analyzed factors on soil enzymatic activity (four-way analysis of variance, ANOVA, at *P* < 0.05)

Variables	Deh	Cat	Ure	Pac	Pal
D	4.732	19.738	21.034	3.812	4.236
S	1.055	16.687	1.710	52.781	72.692
Cs	1.093	5.033	2.307	2.124	0.675
T	79.665	36.758	5.988	9.152	11.201
D × S	0.016	0.073	4.850	2.139	0.702
D × Cs	0.786	6.297	9.841	2.625	0.412
S × Cs	2.855	0.128	0.893	5.962	0.406
D × T	1.232	0.789	1.636	3.940	0.355
S × T	3.824	2.526	18.319	0.111	0.863
Cs × T	1.494	1.553	5.357	0.417	1.468
D × S × Cs	0.251	2.299	6.685	4.769	0.298
D × S × T	0.181	0.281	0.273	1.317	0.540
D × Cs × T	0.518	3.500	9.767	4.408	0.443
S × Cs × T	1.589	0.359	1.798	2.758	0.684
D × S × Cs × T	0.297	1.683	5.271	1.265	0.045
Error	0.412	2.296	4.270	2.422	4.980

*Deh* dehydrogenases, *Cat* catalase, *Ure* urease, *Pac* acid phosphatase, *Pal* alkaline phosphatase*, D* dose of fungicide, *S* type of soil, *Cs* fertilizing substance, *T* date of analysis

**Table 7:** Enzymatic activity in loamy sand, kg^−1^ DM of soil h^−1^

Dose of fungicide (mg kg^−1^)	Dehydrogenases (µMol TPF)		Catalase (Mol O_2_)		Urease (mMol N-NH_4_)		Acid phosphatase (mMol PNP)		Alkaline phosphatase (mMol PNP)	
	Date of analysis (days)									
	19	50	19	50	19	50	19	50	19	50
Soil without fertilizing substance addition										
0.000	3.466c	10.441a	0.232a	0.214a	0.367ab	0.136d	1.405c	1.485bc	0.701d	0.914c
0.166	2.890cd	10.815a	0.225a	0.143c	0.461a	0.330b	1.614ab	1.692a	0.822c	1.123ab
1.660	2.773cd	9.941a	0.216a	0.117d	0.346ab	0.269bc	0.890d	1.691a	0.668d	1.136a
16.60	1.540d	5.764b	0.175b	0.119d	0.344ab	0.154cd	0.876d	1.535abc	0.602d	1.025b
Average	2.667	9.240	0.212	0.148	0.380	0.222	1.196	1.601	0.698	1.050
*r*	−0.948	−0.996	−0.985	−0.492	−0.474	−0.464	−0.643	−0.360	−0.733	−0.101
Lignohumat Super										
0.000	4.294d	9.486b	0.253b	0.182c	0.522ab	0.331bc	1.622ab	1.558ab	0.852b	1.067a
0.166	3.810de	11.05a	0.279a	0.201c	0.578a	0.370b	1.630ab	1.736a	1.194a	1.081a
1.660	3.181ef	10.84a	0.234b	0.133d	0.423ab	0.154cd	1.522ab	1.542ab	0.686c	1.005a
16.60	2.554f	5.913c	0.198c	0.129d	0.367b	0.077d	1.515b	1.495b	0.574d	0.938ab
Average	3.460	9.322	0.241	0.161	0.473	0.233	1.572	1.583	0.827	1.023
*r*	−0.847	−0.940	−0.876	−0.667	−0.792	−0.799	−0.683	−0.589	−0.670	−0.905
Bioilsa N12.5										
0.000	3.676c	12.515a	0.237a	0.161c	0.173c	0.212bc	1.366e	1.674bc	0.950bc	1.142a
0.166	3.435c	11.900a	0.237a	0.179c	0.443a	0.307abc	1.878b	1.645c	0.846cd	1.174a
1.660	2.555c	9.697b	0.225ab	0.212b	0.461a	0.328abc	1.605cd	2.192a	0.773d	1.105a
16.60	2.515c	9.627b	0.208b	0.173c	0.348ab	0.231bc	1.390de	2.385a	0.597e	0.964b
Average	3.045	10.935	0.227	0.181	0.356	0.270	1.560	1.974	0.792	1.096
*r*	−0.663	−0.657	−0.945	−0.164	0.016	−0.393	−0.469	0.796	−0.908	−0.972

*r* Pearson’s linear correlation coefficientHomogeneous groups were marked with the same letters separately for each group of enzymes and the type of fertilizing substance

**Table 8:** Enzymatic activity in sandy loam, kg^−1^ DM of soil h^−1^

Dose of fungicide (mg kg^−1^)	Dehydrogenases (µMol TPF)		Catalase (Mol O_2_)		Urease (mMol N-NH_4_)		Acid phosphatase (mMol PNP)		Alkaline phosphatase (mMol PNP)	
	Date of analysis (days)									
	19	50	19	50	19	50	19	50	19	50
Soil without fertilizing substance addition										
0.000	2.432de	18.449a	0.260a	0.229bcd	0.385a	0.251b	1.408a	1.358ab	1.720e	2.383b
0.166	2.880d	17.720a	0.243ab	0.214d	0.368ab	0.327ab	0.888def	1.190bc	1.485f	2.494a
1.660	2.850d	16.165b	0.236bc	0.170e	0.364ab	0.347ab	0.851def	1.076cd	1.403fg	2.248c
16.60	1.410e	12.247c	0.221cd	0.168e	0.253b	0.308ab	0.762f	1.063cd	1.328g	2.148d
Average	2.393	16.145	0.240	0.195	0.343	0.308	0.977	1.172	1.484	2.318
*r*	−0.936	−0.966	−0.825	−0.659	−0.994	0.064	−0.540	−0.596	−0.665	−0.797
Lignohumat Super										
0.000	2.101e	13.070a	0.273a	0.211c	0.368ab	0.462a	0.569c	1.174a	1.941c	2.231a
0.166	2.532e	11.712b	0.292a	0.234b	0.409ab	0.403ab	0.902ab	1.060a	1.727d	2.103b
1.660	2.357e	10.876c	0.246b	0.214c	0.427ab	0.346ab	0.866abc	1.040a	1.569e	1.973c
16.60	1.191f	7.110d	0.206c	0.198c	0.156c	0.310b	0.611bc	1.036a	1.372f	1.683de
Average	2.045	10.692	0.254	0.214	0.340	0.380	0.737	1.078	1.652	1.998
*r*	−0.945	−0.959	−0.900	−0.744	−0.962	−0.760	−0.448	−0.480	−0.822	−0.928
Bioilsa N12.5										
0.000	2.189b	11.490a	0.274a	0.235cd	0.253d	0.614a	0.923bc	1.238a	2.132b	2.210b
0.166	2.400b	11.530a	0.260ab	0.224cd	0.253d	0.440bc	1.341a	1.028b	1.828cd	2.486a
1.660	2.267b	11.378a	0.237bc	0.216cd	0.272d	0.389bcd	0.903bc	1.011b	1.777de	2.156b
16.60	1.249c	11.032a	0.219cd	0.211d	0.468ab	0.308cd	0.439d	0.850c	1.695e	1.891c
Average	2.026	11.358	0.248	0.222	0.312	0.438	0.902	1.032	1.858	2.186
*r*	−0.983	−0.979	−0.832	−0.725	1.000	−0.716	−0.856	−0.796	−0.619	−0.833

*r* Pearson’s linear correlation coefficient

Homogeneous groups were marked with the same letters separately for each group of enzymes and the type of fertilizing substance

The negative impact of fungicides on soil enzyme activities can be neutralized by adding attenuating substances to the soil, such as compost, manure, basalt meal, or bentonite. Their application to the soil can lead to the stimulation of naturally occurring microorganisms in this environment, thereby, accelerating the rate of fungicide degradation (Adams et al. 2015). In our study, soil supplementation with fertilizing substances had a varied effect on the soil enzymes activity. Their impact was primarily dependent on the type of the soil and enzyme tested (Tables 7, 8, Fig. 3). In loamy sand, Lignohumat Super and Bioilsa N 12.5 preparations caused an increased activity of all enzymes tested. Lignohumat Super increased the activity of dehydrogenases (irrespective of chlorothalonil dose and analysis date) by 7%, that of catalase by 12%, that of acid phosphatase by 13%, and that of alkaline phosphatase by 6% on average, compared to the soil with no addition of fertilizing substances. In turn, Bioilsa N 12.5 increased the activity of dehydrogenases by 17%, that of catalase by 13%, that of urease by 4%, that of acid phosphate by 26%, and that of alkaline phosphatase by 8% on average. The highest content of organic matter contained in the tested compounds helped stimulate enzymes secreted by microorganisms and plants, thereby increasing their count. In addition, organic matter is a catalyst for many biological processes occurring in the soil environment, especially in light soils (Banach-Szott et al. 2014). The effect of these preparations was slightly different in sandy loam. It was observed that the activity of dehydrogenases and acid phosphatase decreased after application of these fertilizers to the soil. Lignohumat Super decreased the activity of dehydrogenases (irrespective of chlorothalonil dose and analysis date) by 31% and that of acid phosphatase by 16%, whereas Bioilsa N 12.5—by 28% in the case of dehydrogenases and by 10% in the case of acid phosphatase. The tested fertilizing substances introduced to sandy loam stimulated activities of catalase and urease. Catalase activity in pots with the addition of Lignohumat Super and Bioilsa N 12.5 preparations increased by ca. 8% on average, whereas urease activity increased by 11% on average upon the addition of Lignohumat Super and by 15% upon the addition of Bioilsa N 12.5. This reduction in enzyme activity could be attributed to the substrate and enzyme adsorption on organic matter surface, resulting in the formation of a substrate-enzyme complex. This in turn caused the inhibition of enzymatic reactions in the soil (Trckova et al. 2004). On day 50 of the experiment, the highest activity was observed for dehydrogenases, acid phosphatase, and alkaline phosphatase both in loamy sand and sandy loam, while on day 19 for catalase activity. Oleszczuk et al. (2014) stated the beneficial impact of biocarbon on the enzymatic activity of the soil treated with dicamba and 2,4-D pesticides.

### Chlorothalonil residues in the soil

**Fig. 4:**
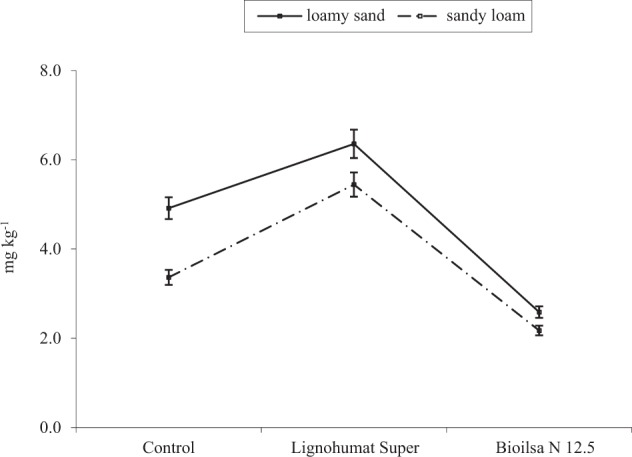
Chlorothalonil residues in the soil applied at a dose of 16.60 mg kg^−1^ (mg kg^−1^ DM of soil)

Despite being so small in size, soil microorganisms are very active in the degradation of pollutants, including fungicides, entering the soil environment (Murali and Mehar 2014). With their participation, the most stable compounds are usually decomposed into less toxic substances. However, the process of degradation can be impaired by lower availability or total disappearance of nutrients that are necessary for the proper functioning of microorganisms (Kanissery and Sims 2011). Biostimulation is a treatment that allows for an enhancement in the rate of fungicide decomposition. The activity of microorganisms naturally occurring in the soil ecosystem (Adams et al. 2015) is stimulated by nutrients (e.g. compost, manure, or straw) introduction into the soil. The results of the current study indicated (Table 9) that the type of fertilizing substance affected the degradation of chlorothalonil the most (up to 85%). In loamy sand without fertilizers (Fig. 4), chlorothalonil was degraded in 70% after 19 days of the experiment, while in sandy loam in 80%. The introduction of Bioilsa N 12.5 into the soil proved to be effective in chlorothalonil transformation in both types of soil. Degradation of chlorothalonil as a result of soil supplementation with this preparation accounted for 84% in the loamy sand and for 87% in the sandy loam. In contrast, Lignohumat Super was ineffective in chlorothalonil degradation. It was observed that chlorothalonil was degraded slower after Lignohumat Super application. The reason for this was a high content of humic substances (90%), which, similarly as loam minerals, are characterized by a high sorption capacity, which is associated with the ability to retain pollutants, such as pesticides. Chlorothalonil sorbed by humic substances became less accessible to microorganisms, and thus was degraded slower in the soil environment (Cycoń et al. 2009). This fungicide strongly bound to humic acids present in the Lignohumat Super preparation, thus it did not undergo desorption and remained longer in the soil environment (Banach-Szott et al. 2014). In loamy sand, the active substance was degraded in 67%, while in sandy loam in 62%. In the study of Singh et al. (2010), the addition of compost into the soil contributed to the fastest transformation of azoxystrobin. However, azoxystrobin was more rapidly transformed in sandy loam than in loamy sand. These studies prove, that degradation of fungicides depends both on their stability in the soil and on soil particle size.

**Table 9:** The percentage of the observed *η*^2^ variability of the effect of the analyzed factors on the degradation of chlorothalonil applied to the soil at a dose of 16.60 mg kg^−1^ DM of soil (two-way analysis of variance, ANOVA, at *P* < 0.05)

Parameter	S	Cs	S × Cs	Error
Residues of chlorothalonil	9.430	84.762	2.217	3.591

*S* type of soil, *Cs* fertilizing substance

### Spring wheat development

**Fig. 5:**
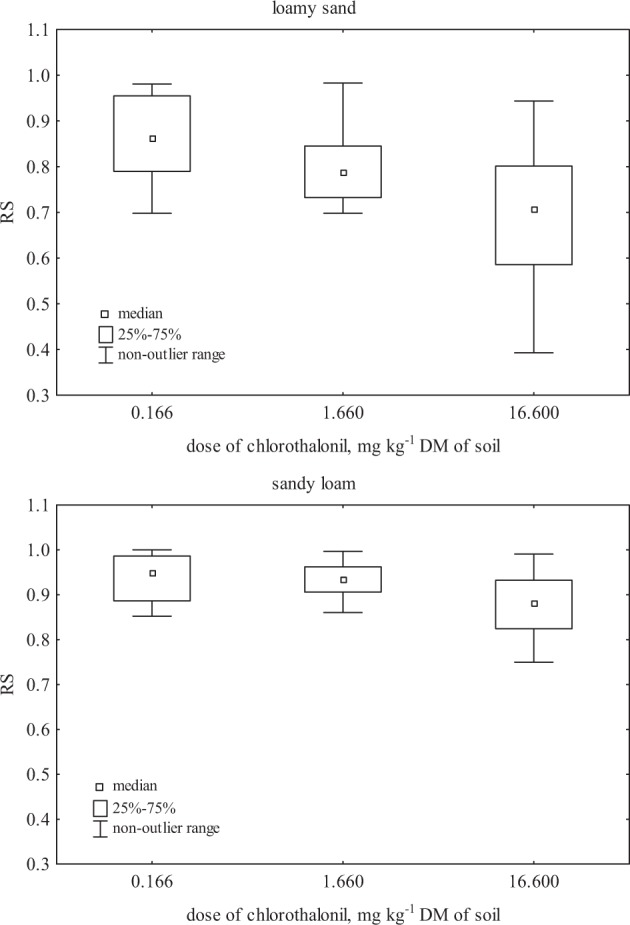
Effect of chlorothalonil on spring wheat resistance (RS)

Three-way analysis of variance ANOVA (Table 10) showed a significant effect of fungicide dose and soil type on spring wheat yield. The dose of chlorothalonil influenced yielding of spring wheat in 39%, and the type of soil in 19%. The introduction of fertilizing substances to the soil had no significant influence on the growth of the tested plant. Fungicides are effective and widely used agricultural chemicals for the control of fungal pathogens in crop plants. However, they can exert a negative impact on plant physiology, mainly through changes in the lipid, protein, chlorophyll or nucleic acid structures. This, in turn, leads to plant growth inhibition and yield reduction (Dias 2012; Petit et al. 2012). The fungicide dose of 16.60 mg kg^−1^ had the highest inhibitory effect on *Triticum aestivum* L., as it reduced its yield by 16% in loamy sand and by 8% in sandy loam (Table 11). The dose recommended by the manufacturer caused no significant changes in plant growth. Spring wheat exhibited varied sensitivity to chlorothalonil, as evidenced by the resistance index (Fig. 5). *Triticum aestivum* L. was the most resistant in the objects with chlorothalonil addition in the dose of 0.166 mg kg^−1^. The mean value of the RS was 0.865 in loamy sand, while 0.946 in sandy loam. The sensitivity of spring wheat increased with fungicide dose, regardless of soil type. However, spring wheat was more resistant to chlorothalonil soil contamination in sandy loam than in loamy sand. This could result from such properties of soil as organic matter content and loam fraction content, and from soil pH, which largely affect pesticides stability in the soil environment. Soils with a higher content of organic matter and the loam fraction are capable to adsorb pesticides in soil colloids, which intensifies their degradation. Pesticides adsorbed by soil colloids are unavailable to root hairs of plant, and thereby are less toxic (Cycoń et al. 2009; Przybulewska and Sienicka 2008). Degradation of pesticides is also more intensive in the soil with pH–7, whereas below this value it is slowed down. In addition, the impact of soil pH on degradation of pesticides depends, to a large extent, on their susceptibility to alkaline or acidic hydrolysis (Shahgholi 2014). Ijaz et al. (2015) studied the effects of triazole fungicides (Torpex, Folicur, Proline, Caramba) and strobilurines (Otrivia, Cantus) on the growth and development of winter oilseed rape. Their results indicated that the proper use of fungicides in accordance with the principles of good agricultural practice could increase yields of winter oilseed rape. Bettiol et al. (2015) observed growth inhibition of *Lepidium sativum* after pesticide applications (chloroxynil, bromoxynil and ioxynil). In the present research, soil enrichment in Lignohumat Super and Bioilsa N 12.5 in order to neutralize the negative effects of chlorothalonil on spring wheat development, proved to be ineffective in increasing crop yield (Table 11). Studies conducted by Adekunle (2011) demonstrated that the adverse effect of pollutants on the condition of the soil environment can be neutralized by the use of biostimulants.

**Table 10:** The percentage of the observed *η*^2^ variability of the effect of the analyzed factors on spring wheat yield (three-way analysis of variance, ANOVA, at *P* < 0.05)

Parameter	D	S	Cs	D × S	D × Cs	S × Cs	D × S × Cs	Error
Yield	39.421	18.791	2.088	8.491	9.858	5.106	6.368	9.876

*D* dose of fungicide, *S* type of soil, *Cs* fertilizing substance

**Table 11:** Spring wheat yield, g pot^−1^ DM of soil

Dose of fungicide (mg kg^−1^)	Loamy sand	Sandy loam
Soil without fertilizing substance addition		
0.000	18.125a	16.600ab
0.166	17.750ab	16.350ab
1.660	16.050ab	16.275ab
16.60	15.275bc	15.325bc
Average	16.800	16.138
*r*	−0.806	−0.982
Lignohumat Super		
0.000	15.850bc	15.375bc
0.166	17.000ab	14.950c
1.660	17.525a	14.725c
16.60	14.975c	14.250c
Average	16.338	14.825
*r*	−0.744	−0.858
Bioilsa N 12.5		
0.000	17.625ab	16.025ab
0.166	17.650ab	16.100ab
1.660	15.600bc	15.750bc
16.60	15.150bc	15.700bc
Average	16.506	15.894
*r*	−0.750	−0.716

*r* Pearson’s linear correlation coefficient

Homogeneous groups were marked with the same letters

## Conclusions

The present study showed that chlorothalonil impacted microbiological and biochemical activity in soil. It stimulated the counts of heterotrophic bacteria and actinobacteria in both loamy sand and sandy loam, while its inhibitory effect was found for fungi. Chlorothalonil also affected soil biochemical properties. It proved to be a inhibitor of acid phosphatase, catalase and dehydrogenase activities. Chlorothalonil exerted variable effects on urease and alkaline phosphatase activities. Spring wheat was also sensitive to excessive quantities of the tested formulation. It should be stressed, however, that the negative effects of the fungicide on soil biological properties were observed in the case of doses many times exceeding the recommended one. Lignohumat Super and Bioilsa N 12.5 fertilizing substances were used to alleviate the negative impact of chlorothalonil on the microbiological and biochemical properties of the soil. Soil supplementation with these preparations had a varied impact on soil biological activity, but they were more effective in loamy sand. Fertilizing substances had no significant influence on the yielding of spring wheat. However, soil biostimulation with Bioilsa N 12.5 accelerated chlorothalonil degradation in the soil compared to the soil without the addition of the fertilizing substance. In contrast, Lignohumat Super was ineffective in degrading the tested fungicide, as the active substance was transformed more slowly after its application than in the control soil. To recapitulate, it may be concluded that irrespective of soil type (loamy sand or sandy loam), chlorothalonil used in the optimal dose causes no changes in the biological homeostasis of soil, while when applied in doses multiply exceeding the optimal dose it disturbs the soil microbiota. Finally, Bioilsa N 12.5 turned out to be more effective in restoring soil fertility than Lignohumat Super was.
